# Application of Machine Learning Using Decision Trees for Prognosis of Deep Brain Stimulation of Globus Pallidus Internus for Children With Dystonia

**DOI:** 10.3389/fneur.2020.00825

**Published:** 2020-08-14

**Authors:** Syed Ahmar Shah, Peter Brown, Hortensia Gimeno, Jean-Pierre Lin, Verity M. McClelland

**Affiliations:** ^1^Usher Institute, Edinburgh Medical School, University of Edinburgh, Edinburgh, United Kingdom; ^2^MRC Brain Network Dynamics Unit, University of Oxford, Oxford, United Kingdom; ^3^Nuffield Department of Clinical Neurosciences, University of Oxford, Oxford, United Kingdom; ^4^Children's Neurosciences Department, Evelina London Children's Hospital, Guy's and St Thomas' NHS Foundation Trust, London, United Kingdom; ^5^Women and Children's Health Institute, King's College London, London, United Kingdom; ^6^Department of Basic and Clinical Neuroscience, Institute of Psychiatry, Psychology and Neuroscience, King's College London, London, United Kingdom

**Keywords:** dystonia, machine learning, deep brain stimulation, decision support systems, decision trees

## Abstract

**Background:** While Deep Brain Stimulation (DBS) of the Globus pallidus internus is a well-established therapy for idiopathic/genetic dystonia, benefits for acquired dystonia are varied, ranging from modest improvement to deterioration. Predictive biomarkers to aid DBS prognosis for children are lacking, especially in acquired dystonias, such as dystonic Cerebral Palsy. We explored the potential role of machine learning techniques to identify parameters that could help predict DBS outcome.

**Methods:** We conducted a retrospective study of 244 children attending King's College Hospital between September 2007 and June 2018 for neurophysiological tests as part of their assessment for possible DBS at Evelina London Children's Hospital. For the 133 individuals who underwent DBS and had 1-year outcome data available, we assessed the potential predictive value of six patient parameters: sex, etiology (including cerebral palsy), baseline severity (Burke-Fahn-Marsden Dystonia Rating Scale-motor score), cranial MRI and two neurophysiological tests, Central Motor Conduction Time (CMCT) and Somatosensory Evoked Potential (SEP). We applied machine learning analysis to determine the best combination of these features to aid DBS prognosis. We developed a classification algorithm based on Decision Trees (DTs) with k-fold cross validation for independent testing. We analyzed all possible combinations of the six features and focused on acquired dystonias.

**Results:** Several trees resulted in better accuracy than the majority class classifier. However, the two features that consistently appeared in top 10 DTs were CMCT and baseline dystonia severity. A decision tree based on CMCT and baseline severity provided a range of sensitivity and specificity, depending on the threshold chosen for baseline dystonia severity. In situations where CMCT was not available, a DT using SEP alone provided better than the majority class classifier accuracy.

**Conclusion:** The results suggest that neurophysiological parameters can help predict DBS outcomes, and DTs provide a data-driven, highly interpretable decision support tool that lends itself to being used in clinical practice to help predict potential benefit of DBS in dystonic children. Our results encourage the introduction of neurophysiological parameters in assessment pathways, and data collection to facilitate multi-center evaluation and validation of these potential predictive markers and of the illustrative decision support tools presented here.

## Introduction

Deep Brain Stimulation (DBS) of the Globus pallidus internus (GPi) is a well-established management for isolated idiopathic or genetic dystonia both in adults (1–4) and children (5). In childhood, acquired dystonias are more common than idiopathic/genetic dystonias, comprising ~80% of patients referred for consideration of DBS (5). There are many reports of successful outcomes in acquired dystonias, but benefits are generally more modest and the variability in outcome is much greater than in idiopathic/genetic dystonias (1, 4, 6). Studies of DBS for acquired dystonia are sparse and generally limited to small numbers. For example, a recent meta-analysis and systematic review of DBS for childhood dystonia yielded individual patient data from a total of only 125 patients with acquired dystonia across 72 articles (7, 8).

Appropriate family counseling is essential to manage expectations before a young person undergoes functional neurosurgery, which is not without risk (9, 10) but predictive markers of DBS outcomes in acquired and complex dystonias are lacking (11) and families are asking for more information to help guide this decision (12, 13).

We have previously reported a relationship between neurophysiological measures of corticospinal tract and sensory pathway integrity and outcome from DBS in a group of children and young people with dystonia (or dystonia-dyskinesia) (6). In that study, abnormalities of either Central Motor Conduction Time (CMCT) or Somatosensory Evoked Potentials (SEP) were associated with less reduction in dystonia at one-year follow-up, as measured using the Burke-Fahn-Marsden Dystonia Rating Scale-motor score (BFMDRS-m) and (for SEPs) using the Canadian Occupational Performance Measure (COPM) (6, 13). This was the first study to investigate the role of neurophysiological tools as potential predictive markers which could help to guide counseling of families (6). However, there were a number of limitations: in particular, although the overall sample was large, the numbers of children with abnormal CMCT and/or SEP who proceeded to DBS and already had 1-year outcome data were small. The current study builds on this previous work by reporting findings from a larger group of young people and by leveraging techniques developed in the machine learning community to investigate the most optimal clinical decision tool that synthesizes various clinical features and assesses their accuracy in predicting outcomes.

## Methods

Data were reviewed retrospectively from all 244 children with medically refractory dystonia who attended King's College Hospital between September 2007 and June 2018 for CMCT and/or SEPs as part of their assessment for possible pallidal Deep Brain Stimulation via the Complex Motor Disorders Service at Evelina London Children's Hospital. This was an extension of a previously published dataset (6) comprising 180 children. The neurophysiological studies were performed as part of a standard clinical work-up, along with detailed imaging and a multi-disciplinary clinical assessment by a pediatric neurologist, nurse specialist, physiotherapist, occupational therapist, speech and language therapist and clinical neuropsychologist. Ethical approval for the retrospective analysis was obtained (London-Harrow National Research Ethics Committee, London, UK (17/LO/0439).

### Data Acquisition

The young people were examined by a consultant pediatric neurologist with expertise in movement disorders (JPL) and dystonia was classified in line with the Albanese dystonia classification (14), taking into account the clinical characteristics and etiology (Table 1). Baseline dystonia severity was assessed using the BFMDRS-m. For those proceeding to DBS (*n* = 133), outcome was expressed as percentage improvement in BFMDRS-m from baseline to 1 year post-operatively. The Canadian Occupational Performance Measure (COPM) was used as an additional outcome measure (13), although was not available in all patients.

**Table 1:** Distribution of patients across different etiological groups.

**Aetiological group**	**Number of patients with satisfactory SEP data (% with respect to all those with satisfactory SEPs)**	**Number of patients with satisfactory CMCT data (% with respect to all those with satisfactory CMCT)**
**Isolated Idiopathic/Genetic** Early onset generalized isolated dystonia. No evidence of structural or degenerative pathology	17 (11.0) (of whom 6 DYT1 positive)	22 (11.5) (of whom 8 DYT1 positive)
**Complex Idiopathic/Genetic** Early onset generalized dystonia with associated features (Normal cranial MRI). No evidence of structural or degenerative pathology	16 (10.3) (of whom 5 DYT11 positive and 1 TIFF1 positive)	21 (11.0) (of whom 4 DYT11 positive and 1 TIFF1 positive)
**Acquired non-degenerative** due to perinatal brain injury (Cerebral Palsy)	70 (45.2)	82 (42.9)
**Acquired non-degenerative** due to metabolic condition	16 (10.3)	19 (9.9)
**Acquired non-degenerative** due to other cause	29 (18.7)	35 (18.3)
**Acquired Degenerative** due to Neurodegeneration with Brain Iron Accumulation (NBIA) or mitochondrial disease	7 (4.5)	12 (6.3)
Total	155	191

*Only the patients with technically satisfactory data are included*.

*155/162 (96%) of children tested for SEPs had technically satisfactory data. 157/191 (82%) of children tested for CMCT had technically satisfactory data*.

**Figure 1:**
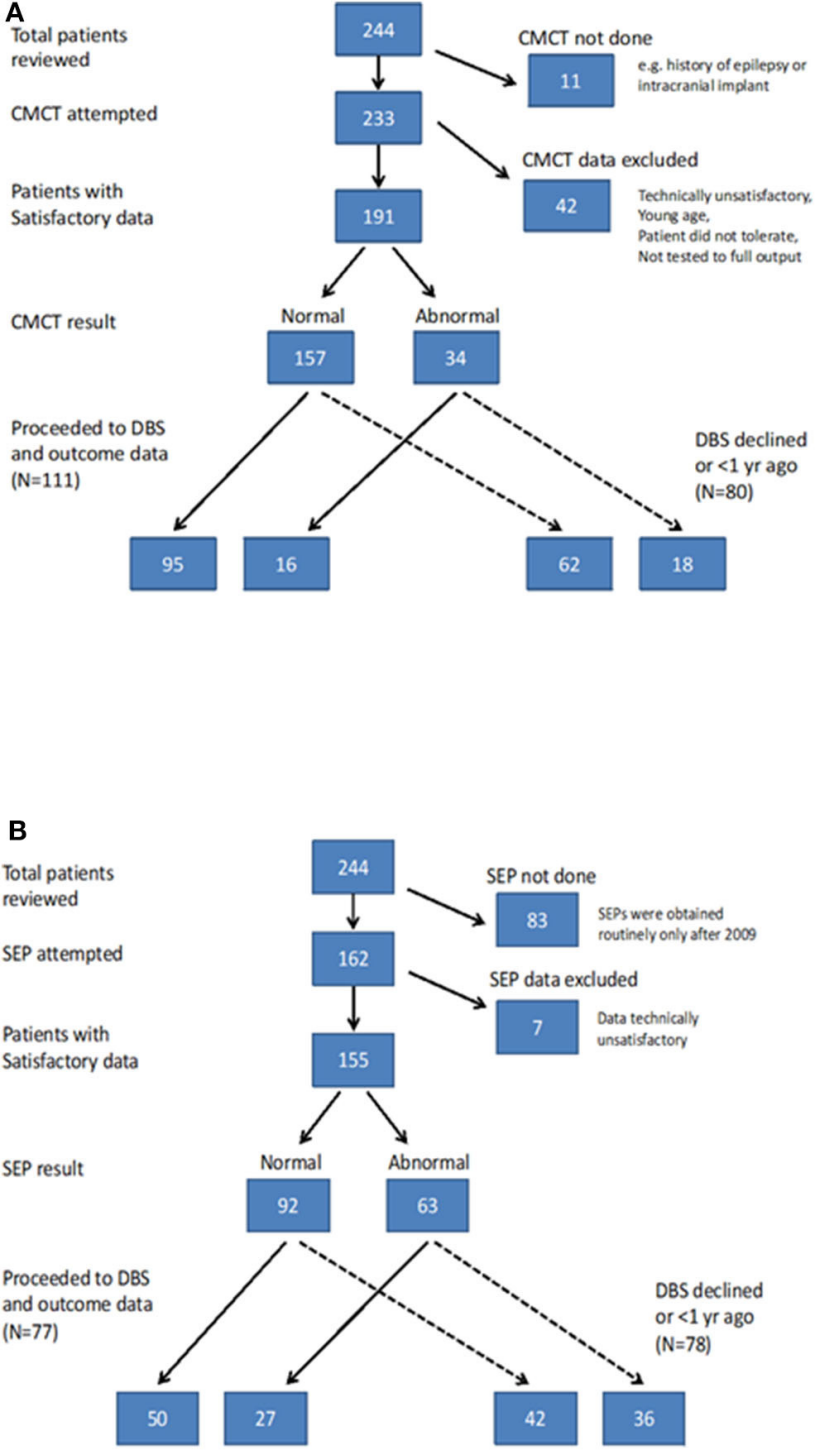
Data flow charts **(A)** CMCT **(B)** SEP.

CMCT was assessed using Transcranial Magnetic Stimulation (TMS) and the F-wave method and interpreted in relation to established norms, as published previously (6, 15). CMCT reaches adult values by age 3 years for upper limbs (16) and by age 6 years for lower limbs (17). A CMCT was considered abnormal if it was prolonged or the MEP to that limb was absent. For the purposes of statistical analysis, any cases in whom a prolonged CMCT was obtained which could have been physiological, due to immaturity, were excluded from the analysis [see (15) for discussion]. Some children with high MEP thresholds were unable to tolerate the stimulus to high enough intensity to determine whether a normal latency MEP was present. These data were excluded along with any other traces which were technically unsatisfactory, as in previous reports (6, 15) (see Flow chart in Figure 1A).

SEPs were obtained from all four limbs, using stimulation of the median nerve at the wrist and posterior tibial nerve at the ankle. Upper limb SEPs were recorded over ipsilateral Erb's point, the 7th and 2nd cervical vertebra and contralateral centro-parietal scalp overlying sensory cortex at C3′ and C4′ (2 cm posterior to C3 and C4). Posterior tibial nerve SEPs were recorded over ipsilateral popliteal fossa and midline scalp at Cz′ (2 cm posterior to Cz) and, more recently (from October 2014 onwards), additionally using a Cz′-Cc derivation (18). The upper lumbar components were recorded where possible (T12/L1—iliac crest). Filter band-pass was 1–500 Hz and the sampling rate 2 kHz. At least two averages of 250 artifact-free trials were recorded to determine reproducibility. The components were labeled according to their polarity and peak-times in adults and the data were compared with published pediatric norms (19–21). Cortical potentials were classed as abnormal if they were delayed (peak times greater than published mean for age-group + 2.5 standard deviations), absent or of abnormal waveform (i.e., clear time-locked cortical activity was present but waveforms were poorly formed or broadened) (see Figure 1). Technically unsatisfactory recordings were excluded from further analysis (see Flow chart in Figure 1B).

For each individual, there was a maximum of 4 limbs of SEP data and 4 limbs of CMCT data, but not all individuals had satisfactory data recorded from all 4 limbs. To simplify the analysis, a “binary coding” was assigned to the SEP and CMCT data for each child. Thus, if CMCT to one or more limbs was abnormal, that child was considered in the “abnormal CMCT” group. Likewise, if the cortical SEP from one or more limbs was abnormal, that child was considered in the “abnormal SEP” group, corresponding with previous reports (6).

MR examination was performed on an Achieva 1·5 Tesla MRI system (Philips, Best, Netherlands), under general anesthesia. Images were acquired using an 8-channel head coil, according to the local “DBS protocol,” to include those sequences required by the neurosurgeons for electrode targeting in the event that the patient went forward for DBS surgery (6, 15). The MRI scans were interpreted by a consultant neuroradiologist and the findings classified, for the purposes of this study, on the anatomical location of abnormalities (6, 15) with the rationale to identify abnormalities in the target nucleus (globus pallidus internus) and to identify patterns of imaging abnormality which would be expected to be associated with dysfunction of the Corticospinal tract (Table S-1 and Figure S-2).

#### Neurosurgical Procedure

Surgery was performed under isofluorane general anesthesia, in view of the young age of the children. Stereotactic MRI was performed pre-operatively under anesthesia with a Leksell G Frame in place to determine co-ordinates targeted in the postero-latero-ventral GPi. Bilateral electrodes were implanted in each case. The electrodes used are all Medtronic 3389 circumferential electrodes: contacts 0.5 mm apart and 1.5 mm in length. Final electrode placement was confirmed by post-operative stereotactic CT scan, under the same general anesthetic, fused with the intra-operative in-frame pre-surgical MRI. The pulse generator was then inserted (Soletra and Kinetra until 2008, and Activa RC pulse generators thereafter, Medtronic, Minneapolis, MN, USA).

Accuracy of electrode placement within our service has been studied previously (22). Mean Euclidean distance between final electrode tip position and target position was 2.2 mm with no difference in accuracy between isolated genetic/idiopathic and acquired dystonia cases. No correlation was found between outcome at 1 year and Euclidian distance between target and actual position (22).

#### Analysis of Imaging and Neurophysiology Parameters in Relation to Outcome

Of the 244 children, 133 (54.5%) went forward for DBS and had 1-year outcome data available. All these children had cranial MRI, 111 (83.4%) had satisfactory CMCT data and 77 (57.8%) had satisfactory SEP data (Figure 1) [Note SEP recordings were incorporated into the assessment pathway more recently than CMCT, hence the lower numbers (see Figure 1B)]. Baseline statistical analysis of these parameters in relation to outcome was performed in SPSS, as per McClelland et al. (6), to allow comparison with the previous report. Differences between groups were investigated using Mann-Whitney test for percentage change in BFMDRS-m (non-normally distributed data) and independent samples *t*-tests for COPM scores (normally distributed data) (see Supplementary Material).

The main purpose of the current report is to investigate the potential application of a Machine Learning approach to the prediction of outcome from DBS based on the following parameters: sex, etiology, baseline severity, cranial MRI, CMCT, and SEP. Data from all children proceeding to DBS and with 1-year outcome data (*n* = 133) were included in the Machine Learning analysis, including initially those who had outcome data, but in whom no satisfactory CMCT or SEP data was available. For the purpose of the ML analysis CMCT was classified as Normal/Abnormal/Not available (NA) and SEP was classified as Normal/Abnormal/NA.

### Machine Learning for Clinical Decision Support

With the large increase in the amount of data that is now collected, and increasing computational power, a large suite of techniques [commonly termed as “Machine Learning” (ML)] have been developed that have led to disruptive changes in a wide variety of industries. Unlike other industries, adoption of ML-based solutions in routine clinical practice is slow due to the unique challenges of healthcare delivery. One such challenge is the need for clinically interpretable algorithms. Many ML techniques are black boxes which are not suitable, especially in a clinical problem setting where existing evidence is sparse. It is therefore important that any ML-based algorithm developed is clinically interpretable. One of the most widely explored fields within ML, and the one that is most relevant to clinical decision support systems, is supervised learning.

#### Supervised Learning

**Figure 2:**
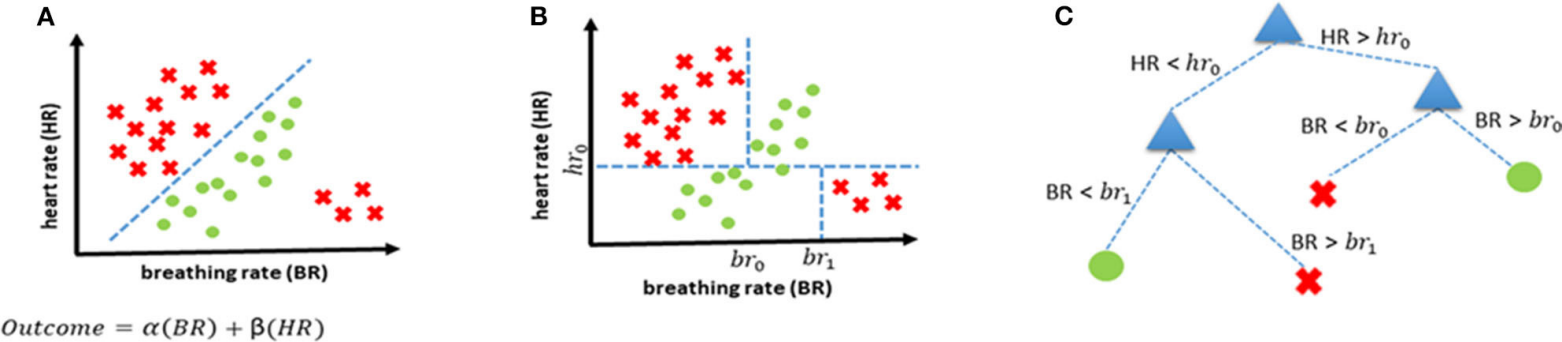
Examples of decision boundaries and trees. **(A)** Linear decision boundary for classification, and the corresponding linear function representing the straight line that separates the two classes. **(B)** Non-linear decision boundary achieved using a decision tree. **(C)** Decision tree corresponding to the decision boundary shown in **(B)**.

Supervised learning uses previous examples with known outputs (or labels) to determine the most optimal decision boundary that can then be used to classify unseen data. This can best be explained with the help of Figure 2A which shows two classes (red crosses, and green circles) and the classification task is to determine a decision boundary that can help identify if a new case belongs to the red class or the green class. A linear classifier will seek to determine a straight line that can best separate the two classes. Mathematically, this is equivalent to finding the values of α and β (through optimization using algorithms such as gradient descent) of a linear equation (α(*BR*)+ β(*HR*) ) that leads to the most “optimal” classification. There are several ML-based classification algorithms, each with their own optimality criterion. Logistic regression (LR) is one of the most commonly used methods for supervised classification. LR is generally simple, easy to implement and interpretable. There are, however, two drawbacks of using an LR-based classification in our case. Firstly, it is a linear classifier, attempting to find a linear combination of different predictors. The example in Figure 2 shows a situation where a linear classifier will not be able to correctly classify all cases as the separation between classes is non-linear. For LR-based classification algorithms, such cases can be handled by introducing new, non-linear features but this increases the chances of over-fitting. Secondly, LR is not ideally suited to using categorical features and requires techniques such as hot-encoding (a machine learning technique that converts categorical features into numerical values so that algorithms can work as intended, but which may also increase the chances of overfitting) (23).

#### Decision Trees

Consequently, we propose using decision trees (DT) as the most appropriate supervised classification method in our case. DTs offer the possibility of combining features non-linearly thereby enabling more complex boundaries to be drawn in the feature space. They also handle categorical features without the need for any hot-encoding. Lastly, they are easily interpretable as every decision can be precisely explained. The same scenario that was introduced earlier in Figure 2A is shown to be handled more elegantly with a decision tree-based classifier (Figure 2B) and the corresponding decision tree is shown in Figure 2C.

In the current study, up to six possible clinical features (sex, etiology, baseline severity, cranial MRI, CMCT, and SEP) were investigated in order to determine the most useful combination for predicting DBS prognosis (favorable or unfavorable).

#### Training, Validation, and Performance Evaluation

In this study, we used k-fold cross validation. This method is used routinely as an internal validation technique where the data are divided into k groups (each patient's data is randomly allocated in one of the k groups) and the algorithm is then trained on the data from all groups except one. The trained algorithm is then tested on the group that was not part of the training set (i.e., out-of-sample testing). This process is repeated k times. This effectively allows us to use all the data for testing while ensuring that the same data is not used for both training and testing to avoid over-fitting. The metrics we used to assess the performance of the algorithms are based on True Positives (TP: number of patients with favorable prognosis that are correctly predicted), True Negatives (TN: number of patients with poor prognosis that are correctly predicted), False Positives (FP: number of patients with unfavorable DBS outcomes who were wrongly predicted to have a favorable prognosis), and False Negatives (FN: number of patients with favorable DBS outcomes who were wrongly predicted to have an unfavorable prognosis) and these are:

$$\begin{array}{l} Accuracy=\frac{TP+TN}{TP+TN+FP+FN}\\ Sensitivity=\frac{TP}{TP+FN}\\ Specificity=\frac{TN}{TN+FP} \end{array}$$

In order to benchmark the performance of the algorithms, we defined a majority class classifier as the reference. A majority class classifier always outputs the class that is in majority irrespective of the input. The accuracy of such a classifier will be equal to the proportion of the majority class, and any classifier that results in an accuracy greater than the majority class is deemed good. For example, if 48 out of 80 patients show a positive response to a treatment (without taking into account the variables in question), then the majority (60%) of patients improve and the majority class classifier performance is defined as 60% (i.e., one would predict that 60% of patients would show a positive response). The aim of the analysis is to test whether any additional classifiers improve the accuracy of the outcome prediction above this baseline level.

We also used receiver operating characteristics (ROC), where applicable, to determine a range of sensitivity and specificity values as the threshold (BFMDRS-m score in this case) for decision making is changed. As the overall accuracy of any decision tree on the test set (i.e., out-of-sample accuracy) is dependent on the allocation of patient data in various groups during k-fold cross validation, we used multiple iterations and then computed the mean of the resulting accuracy, and where applicable, we have also reported the error bounds (one standard error) of our estimates.

Considering the six clinical feature variables (sex, etiology, baseline severity, cranial MRI, CMCT and SEP), we investigated all possible combinations of these six variables to construct decision trees (DT) and evaluated the corresponding performance with k-fold cross validation. In total, there were (2^6^−1) i.e., 63 different combinations that were investigated.

All the analysis was carried out in MATLAB (24). For fitting a decision tree model, we used the “fitctree” provided in MATLAB. The hyperparameters in the algorithm were learnt using “Bayesian Optimization.”

## Results

### Standard Feature-Outcome Associations of Imaging and Neurophysiology Parameters

The results of CMCT and SEP data for the cohort as a whole are in keeping with the previously reported smaller dataset (6) and are reported in the Supplementary Material. Of the 244 children, 133 went forward for DBS and had 1-year outcome data available. All these children had cranial MRI, 111 had satisfactory CMCT data (compared with 89 in previous cohort) and 77 had satisfactory SEP data (compared with 51 in previous cohort) (6). Statistical analysis of these parameters in relation to outcome was also concordant with the previously reported smaller dataset and is shown in Figures S-3, S-4. The remainder of the results focus on the ML analysis.

### Machine Learning Analysis

**Figure 3:**
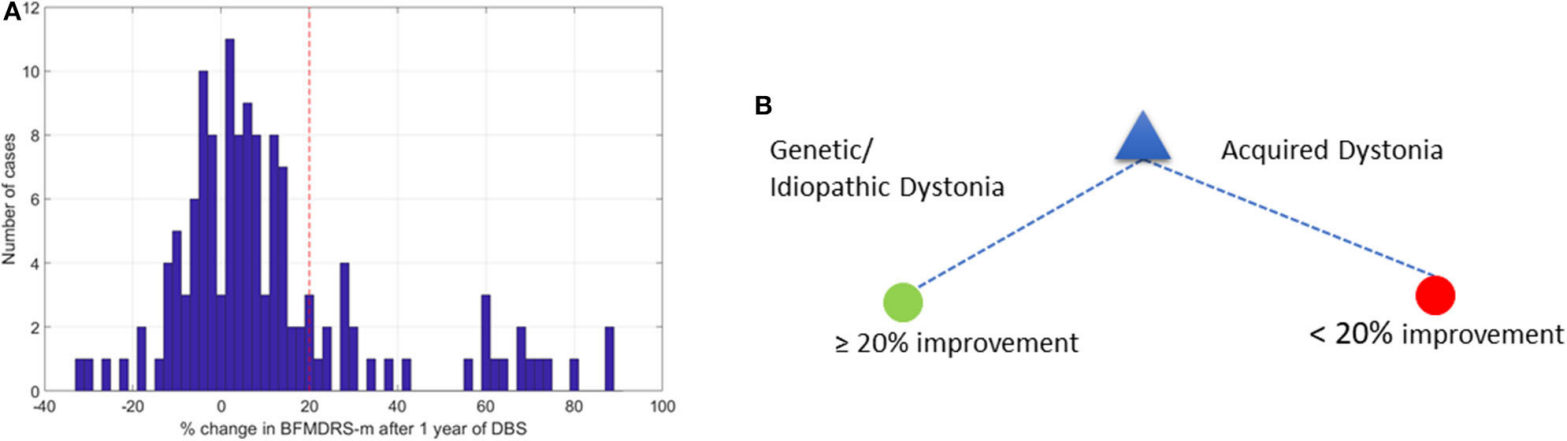
Change in BFMDRS-m after 1 year and decision tree with 20% cutoff. **(A)** Distribution of % change in BFMDRS-m across all patients, with 20% improvement marked by the red vertical line. **(B)** Decision tree based on cut-off of ≥20% improvement in BFMDRS-m, suggesting that DBS prognosis is favorable when patients have genetic or idiopathic dystonia, and less favorable in cases with acquired dystonia.

Figure 3A shows the overall distribution of percentage improvement in BFMDRS-m after 1 year follow-up across the 133 patients. In literature pertaining to DBS outcomes, a ≥20% change in BFMDRS-m at 1 year has been reported as a cut-off for defining improvement (25, 26), although this scale was not developed for use in children (8) and is of limited use in acquired dystonia or dyskinetic cerebral palsy (13, 27, 28). In the current dataset, which is dominated by patients with acquired dystonia, the majority of patients do not reach this level of change. However, we initially investigated which DT leads to the highest accuracy based on this criterion, to allow comparison in the context of the wider literature.

Figure 3B shows the optimal DT that was determined. This suggests that etiology alone can provide the highest accuracy when determining whether DBS can lead to ≥20% change in BFMDRS-m at 1 year. According to this DT, any patient who has an isolated genetic or idiopathic dystonia or a complex genetic or idiopathic dystonia (see Table 1) is likely to benefit from DBS. In this case, the majority class classifier had an accuracy of 80.5% (i.e., 80.5% of the patients will not achieve ≥20% change in BFMDRS-m). Using the DT resulted in out-of-sample accuracy of 85.5% (an improvement over the majority class classifier).

As noted above, there is already good evidence that individuals with isolated genetic or idiopathic dystonias (previously termed primary) are likely to show improvement with DBS, including a recent meta-analysis of DBS for dystonia in children (8). The area in which predictive factors are particularly needed, however, is with respect to acquired dystonias, in whom the degree of benefit is more variable between individuals and harder to predict (8, 15, 29). Apart from a small study in 10 patients with dystonic-dyskinetic cerebral palsy (28), the above 20% improvement cut-off has not been validated in pediatric studies. Previous studies have suggested that improvements in BFMDRS-m more modest than 20%, are still beneficial to patients and their families (4, 13, 27, 30). Relatively small reductions in dystonia can bring meaningful benefit in function and quality of life (see discussion later on other scales).

**Figure 4:**
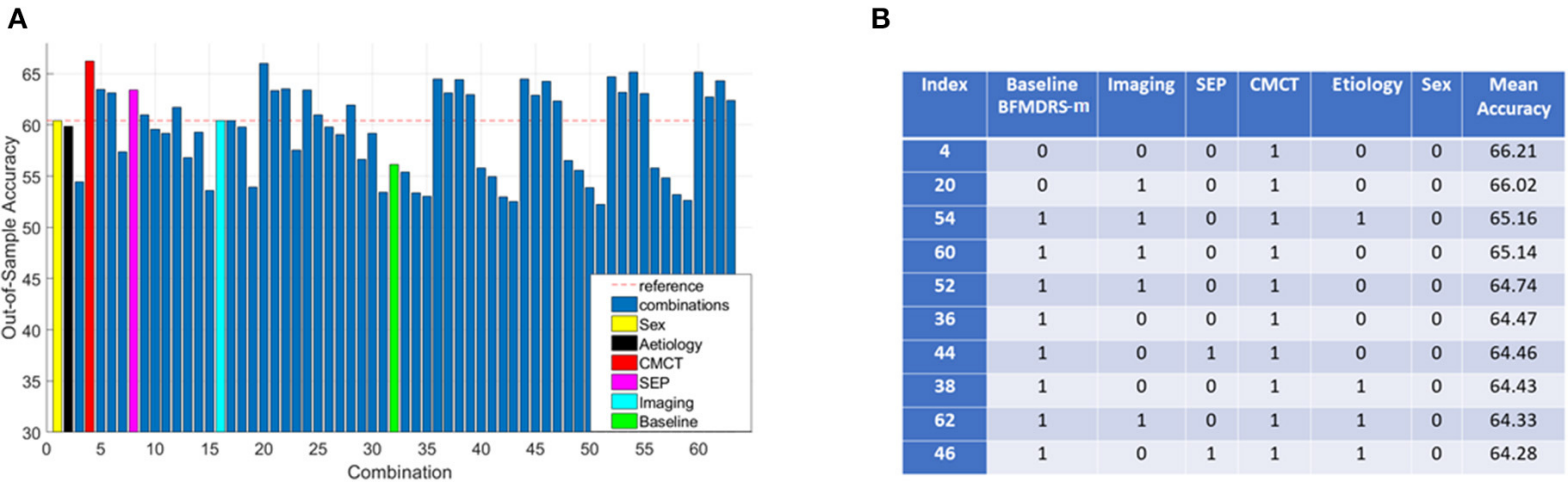
Decision tree performance and winning tree with cut-off ≥0% improvement in BFMDRS-m considering all possible combinations. **(A)** Out-of-sample accuracy of decision trees investigating all possible combinations (63) for patients with acquired dystonia, reference accuracy corresponding to the percentage of majority class, and decision trees based on single feature highlighted, suggesting that CMCT is the most informative feature. **(B)** The top 10 trees, based on accuracy, with the index number associated with those in **(A)**. Each possible combination in the table is binary-coded (1 indicating inclusion of the feature, and 0 indicating the feature was not included in that combination) and the associated accuracy is reported (minimum leaf size of 10, i.e., the minimum number in end nodes, 100 iterations, 10 fold cross validation).

Consequently, further investigation focused on the 96 acquired dystonia cases, and any improvement, defined as >0% change in BFMDRS-m was considered as a positive outcome while the remaining cases were considered as negative (other thresholds were also investigated as described later). The majority class classifier for this analysis had an accuracy of 60.42% (i.e., 58 of the 96 children with acquired dystonia proceeding to DBS had a positive outcome by this definition). Figure 4A shows the overall accuracy after investigating all 63 possible combinations of clinical variables (sex, etiology, baseline severity, cranial MRI, CMCT, and SEP) and a corresponding table listing the top 10 best performing combinations based on classifier accuracy. It is worth noting that as the assigning of patients in k groups during k-fold cross validation is random, the overall accuracy will vary each time the k-fold cross validation procedure is repeated. Figure 4A therefore shows the mean (over all iterations) and Figure 4B lists the top 10 best performing combinations based on mean accuracy. Looking at performances using individual features, we can see that only a decision tree using CMCT or SEP (as an individual feature) outperforms the majority class classifier accuracy, yielding accuracies of 66.21 and 63.39%, respectively.

Because the performance of any decision tree depends on which data samples are assigned in which folds (the smaller the data size, the more likely it is to have results sensitive to which folds data are assigned to), we decided to consider the top 10 best performing decision trees as opposed to picking a single best performing tree. These 10 combinations performed fairly similarly, and all have CMCT present as a feature. We can also see baseline dystonia severity is the second most common feature that most consistently appears (along with CMCT) in the top 10 best performing combinations. We therefore chose the decision tree (index 36) that uses CMCT and baseline dystonia severity for further analysis, for two reasons. Firstly, both CMCT and baseline severity are the two features that appear the most consistently in the top 10 best performing combinations. Secondly, baseline severity is a continuous variable which then allowed us to demonstrate how we could select a cut-off threshold and its implication on sensitivity and specificity for personalized clinical decision making.

**Figure 5:**
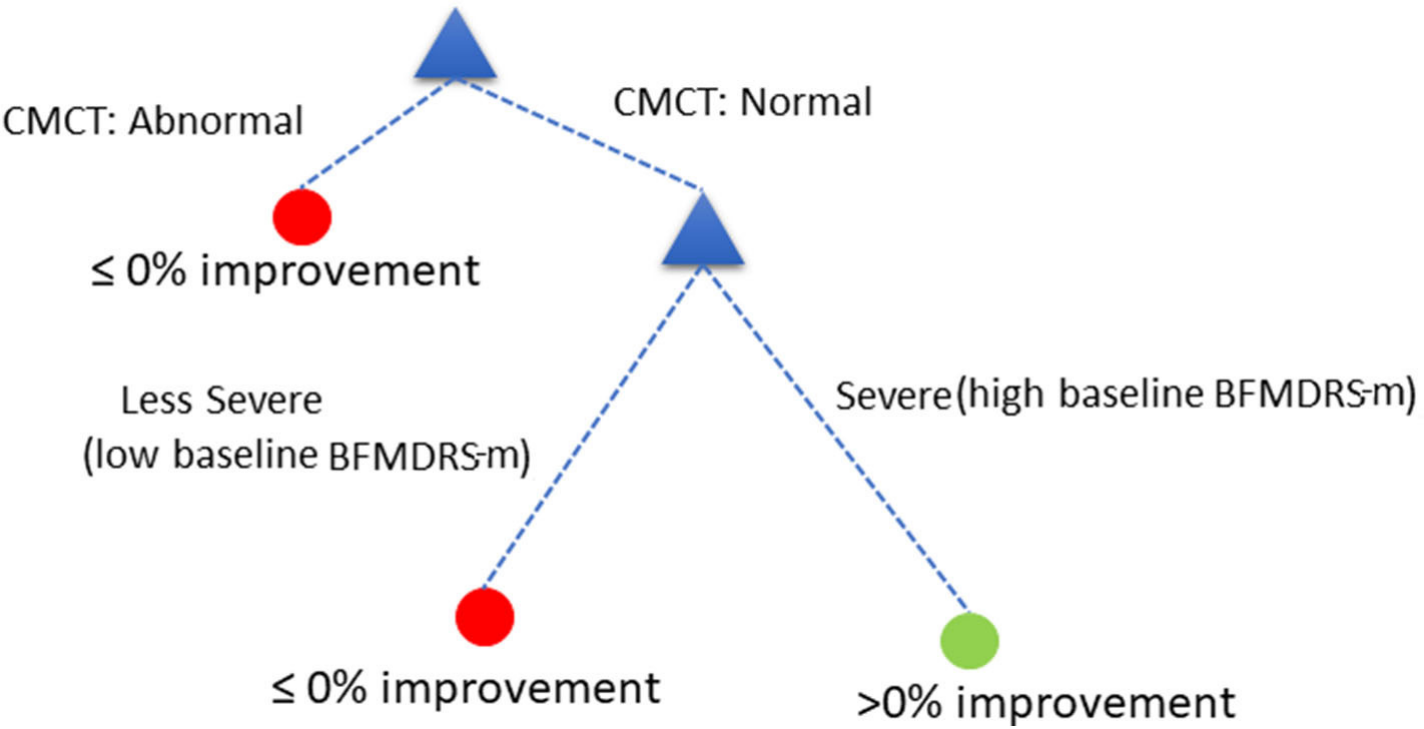
Decision tree based on cut-off of ≥0% improvement in BFMDRS-m, that uses CMCT and baseline severity.

The decision tree showed equivalent performance regardless of the order in which the variables were included. For the purposes of illustration, we used the following DT (Figure 5):

Assess child by CMCT. If CMCT is abnormal, DBS is less likely to be effective at reducing dystonia. If CMCT is normal, assess the child's baseline BFMDRS-m to determine severity. If the child's condition is very severe (based on a specific cut-off chosen automatically by the decision tree—see below), DBS is more likely to be effective in reducing dystonia severity, as measured using BFMDRS-m. For children with lower baseline severity (i.e., less than the threshold identified automatically), DBS is less likely to be effective in reducing dystonia, as measured using BFMDRS-m.

**Figure 6:**
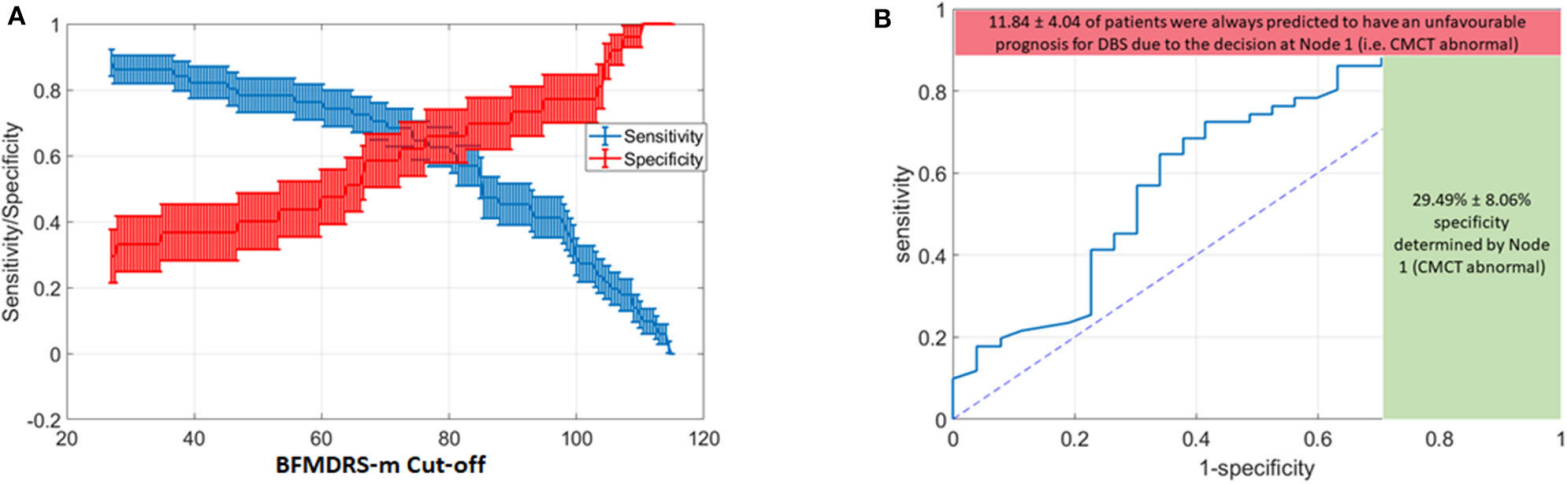
Variation in Decision tree performance with different cut-offs for baseline severity and ROC curve for baseline severity cut-off at point of transection of specificity and sensitivity curves **(A)** Sensitivity and Specificity for different thresholds on BFMDRS-m at Node 2, after applying the decision tree based on CMCT and BFMDRS-m on patients using Monte Carlo simulation. **(B)** Corresponding ROC curve which also shows the upper bound on sensitivity (88.16%), and lower bound on specificity (29.49%) determined by the decision at Node 1 (i.e., CMCT abnormal).

However, severity is a continuous variable, so what value should be chosen as a cut-off? As the BFMDRS-m cut-off used in the DT is varied, so will the resulting accuracy and the likelihood of missing a positive effect. We consequently devised a Monte Carlo simulation technique to help inform case-specific decisions given patient, family and clinical preferences and the current data (see Figure 5). Furthermore, we removed all the cases where CMCT data were unsatisfactory (18 cases out of the total of 96 cases with acquired dystonia) to remove any potential bias in results (see Discussion). In the Monte Carlo simulation, we sampled 100 cases (with substitution) from the total pool of acquired dystonia patients (78 cases after removing those who did not have CMCT available) for 1,000 times and computed the sensitivity and specificity for every possible threshold of the BFMDRS-m in steps of 0.5. Figure 6A shows the resulting sensitivity and specificity plotted with error bars corresponding to one standard error and Figure 6B shows the associated ROC curve. From the figure, it is obvious that there is a trade-off between sensitivity and specificity. Some working examples taking specific baseline severity cut-offs are as follows:

- If one sets the cut-off baseline severity BMFDRS-m score at 80, then patients with a baseline score of >80 would be predicted by the DT to have favorable prognosis from DBS while those with baseline score of <80 would be predicted to have less favorable prognosis. The **sensitivity** at this cut-off is 0.62, which means that 62% of those with a positive outcome would be correctly identified by this DT, while 38% of those who could have had a positive outcome with DBS would be predicted wrongly to have a poor outcome. The **specificity** at this cut-off is 0.64 which means that 64% of those with a poor outcome would be correctly identified by the DT as having a poor prognosis (true negatives), whereas 36% of children predicted to have a good outcome by the DT would actually have a poor outcome (false positives).
- If one sets the cut-off baseline severity BMFDRS-m score at 100 then patients with a baseline score of >100 would be predicted by the DT to have favorable prognosis from DBS while those with baseline score of <100 would be predicted to have less favorable prognosis. The specificity at this cut-off is 0.78, which means that 78% of those predicted to have a positive outcome would do so and there would be fewer (22%) false positives. However, the sensitivity at this cut-off is 0.28, which means that only 28% of those with a positive outcome would be correctly identified by this DT and 72% of those who could have benefitted would be predicted wrongly to have a poor prognosis.
- If one sets the cut-off baseline severity BMFDRS-m score at 60 then patients with a baseline score of >60 would be predicted by the DT to have favorable prognosis from DBS while those with baseline score of <60 would be predicted to have less favorable prognosis. The sensitivity at this cut-off is around 0.75, which means that 75% of those with a positive outcome would be correctly identified by this DT, with fewer (25%) false negatives (those who *could* have had a positive outcome with DBS but were predicted wrongly to have a poor outcome). The trade-off is that the specificity at this cut-off is around 0.45, which means that only 45% of those predicted to have a positive outcome would do so and there would be more (55%) false positives.

Increasing the cut-off of BFMDRS-m makes it less likely that a patient will get a positive prognosis leading to increased specificity at the cost of decreased sensitivity. Thus, if the patient, family and clinician together feel that they would rather not miss the possibility of a positive surgical outcome, they could opt for a BFMDRS-m cut-off to the left, and the corresponding sensitivity and specificity of the outcome prediction considered. Conversely, if the patient, family and clinician together take a more risk adverse view and want to be more certain of the predicted outcome then the BFMDRS-m cut-off might be taken to be to the right, and the corresponding sensitivity and specificity considered in coming to a final decision.

In summary, the range of sensitivity/specificity in the figure will vary according to threshold selected for BFMDRS-m at the second node of the decision tree. However, the decision at node 1 (whether CMCT is normal or abnormal) results in the red and green bounds shown in Figure 6B. On average, there were 11.84% of patients who, despite favorable DBS outcome, had an abnormal CMCT. If this decision tree was to be used for clinical decision making, then 11.84% of cases who could have benefited from DBS would instead be deemed to have a poor DBS prognosis. As sensitivity captures the proportion of patients with favorable DBS prognosis who are correctly identified, missing these 11.84% cases results in the upper bound on sensitivity shown in red. However, at the same time, 29.49% of patients with unfavorable DBS outcomes are correctly identified at node 1. As specificity captures the accuracy of identifying the proportion of people with unfavorable DBS outcomes, there were 29.49% of such patients with an abnormal CMCT (shown by the green bound on specificity) and hence would be correctly deemed to have an unfavorable DBS prognosis (based on these criteria).

**Figure 7:**
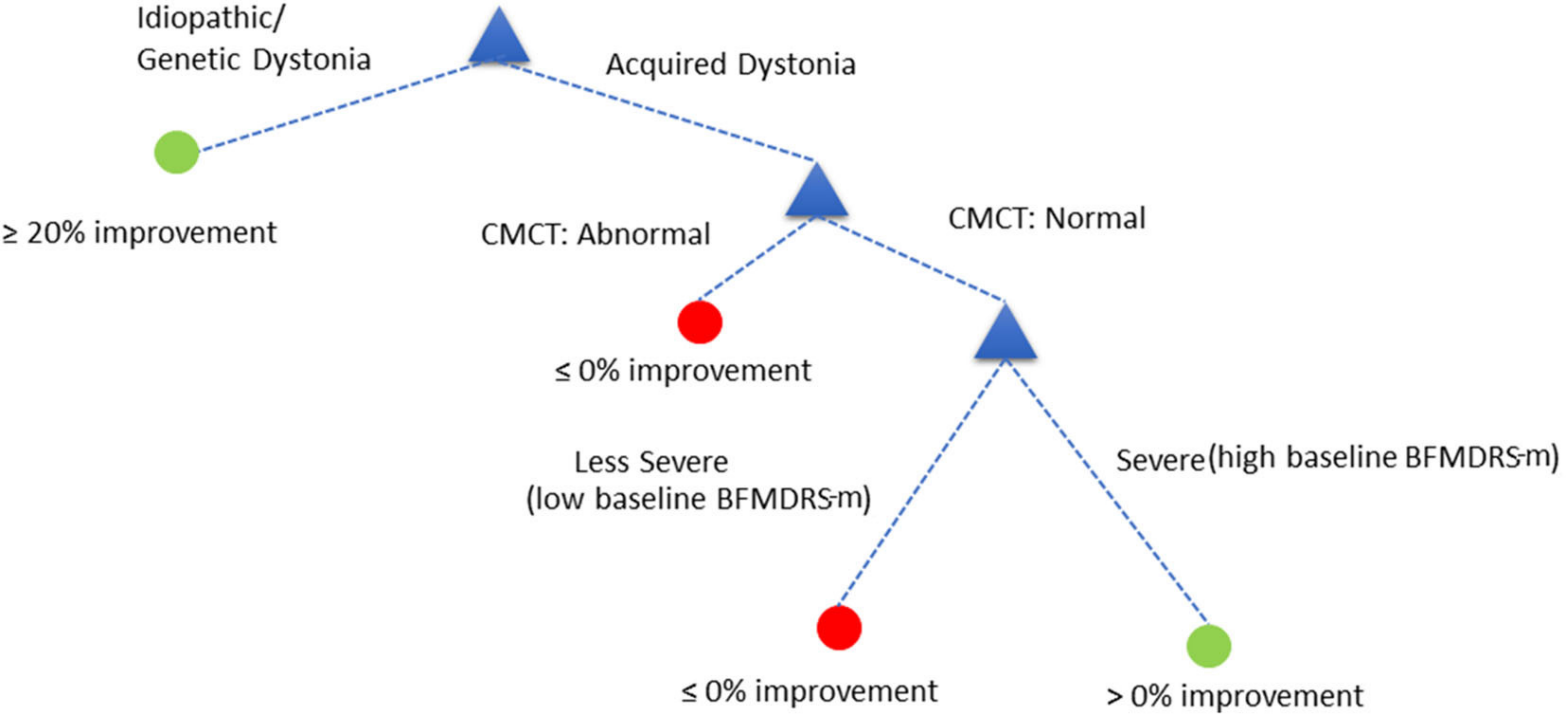
Overall decision tree to determine if DBS will have favorable prognosis.

Figure 7 proposes the overall decision tree that combines the analysis of the whole population (when using a threshold of ≥20% change in BFMDRS-m as improvement) and the analysis carried out on cases with acquired dystonia (when using a threshold >0% change in BFMDRS-m as improvement).

**Figure 8:**
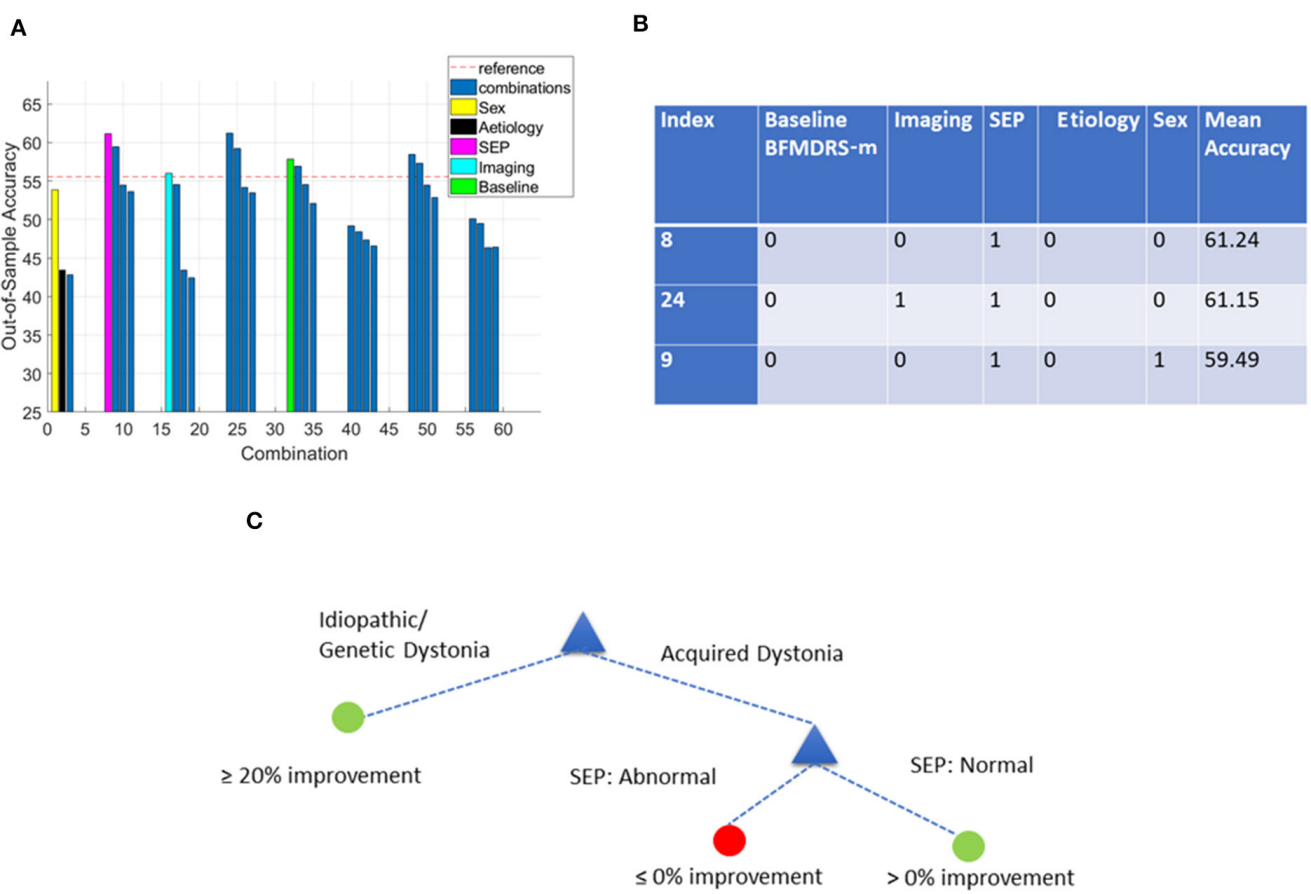
Decision tree performance and winning tree with cut-off ≥0% improvement in BFMDRS-m considering all possible combinations except CMCT. **(A)** Out-of-sample accuracy of decision trees investigating all possible combinations (31) for patients with acquired dystonia, reference accuracy (55.56%) corresponding to the percentage of majority class (based on the 54 patients with SEP data), and decision trees based on single feature highlighted. **(B)** The top three trees, based on accuracy, with the index number associated with those in **(A)**. Each possible combination in the table is binary-coded (1 indicating inclusion of the feature, and 0 indicating the feature was not included in that combination) and the associated accuracy is reported. **(C)** Overall decision tree found for cases with acquired dystonia when CMCT is presumed unavailable.

Since TMS is not available in all centers and, as we report above, is sometimes not performed for clinical reasons, or cannot be completed, we also looked at the data from the viewpoint of CMCT not being done. There were 54 patients with acquired dystonia who had SEP performed. We analyzed this cohort of 54 patients using a similar approach as before, testing all possible combinations (but without CMCT this time). Figure 8A shows the overall accuracy after investigating all 31 possible combinations of clinical variables (sex, etiology, baseline severity, cranial MRI, and SEP). Compared with Figure 4, fewer DTs are seen to exceed the majority class classifier. However, several trees did lead to improved accuracy and the top 3 best performing combinations are listed in Figure 8B. These results suggest that SEP is an important factor for predicting DBS prognosis for a child with acquired dystonia in cases where CMCT is not present. However, no other feature (cranial MRI, sex, etiology, and baseline severity) provides any further predictive value in this analysis. Figure 8C shows the resulting decision tree in cases where CMCT is not available.

Furthermore, given that inter-rater reliability for scoring BFMDRS-m is not 100%, we also investigated the impact of choosing different thresholds of the BFMDRS-m to define positive and negative surgical outcomes in acquired dystonia. Table 2 summarizes the number of cases with positive and negative outcomes, and features that appeared in the best performing models (i.e., those that appeared in >5 models out of the top 10 models). While the results vary due to the limited number of cases, CMCT appears as one of the best features for all possible thresholds of BFMDRS-m that were investigated.

**Table 2:** Variation of number of positive/negative cases in acquired dystonia, and the top features (those that appear in >5 top 10 models) as the threshold on BFMDRS-m to define improvement/no improvement is varied.

**Threshold on**	**Improved**	**Not Improved**	**Best features (i.e.**,
**% change in**	**(%)**	**(%)**	**appeared >5**
**BFMDRS-m**			**models out of the**
			**top 10 models)**
0	58 (60.4)	38 (39.6)	CMCT, Baseline Severity
1	58 (60.4)	38 (39.6)	CMCT, Baseline Severity
2	52 (54.2)	44 (45.8)	CMCT, Etiology
3	48 (50.0)	48 (50.0)	CMCT
4	45 (46.9)	51 (53.1)	CMCT, Baseline Severity
5	41 (42.7)	55 (57.3)	CMCT, Sex
>5	Performance degradation (not enough models that perform better		
	than majority class classifier)		

Lastly, given the limitations of using BFMDRS-m in a pediatric population (8), especially in acquired dystonia and dyskinetic cerebral palsy (13, 27, 28), we also investigated the use of COPM to separate positive DBS outcomes from negative outcomes. The number of patients who had COPM data was less than in the original group of patients with post-operative results (96 vs. 133). A change in COPM score of ≥2 is considered clinically significant (13), so in our preliminary analysis, we used this threshold to separate positive from negative DBS outcomes. However, using these data, we were not able to find any decision tree that performed better than the majority class classifier.

## Discussion

We investigated the prognostic value of six key pre-surgical clinical features (sex, etiology, baseline severity, cranial MRI, CMCT, and SEP) in a cohort of 133 children progressing to DBS for dystonia. Concurring with previous reports, the clearest distinction in outcome was between those children with genetic or idiopathic dystonias, compared with acquired dystonias. Focusing on the 96 children with acquired dystonia, we found that CMCT is the feature with greatest value in predicting improvement after surgery, and where this test is not available or technically unsatisfactory, then SEPs offer an alternative source of prognostic information. Based on these findings we suggest a data-driven, clinically useful tool as an illustration of how ML techniques could support the decision whether or not to proceed to DBS in young people with dystonia (Figures 7, 8). The decision support tool is the product of decision tree analysis, and the sensitivity and specificity of the predictions underpinning the tool are specified. In addition, we suggest a novel method whereby decisions can accommodate case-specific preferences. Such ML-based decision support tools should be revised as necessary as more data become available, and we emphasize that our primary goal here is to use the present cohort to illustrate how ML could support clinical decision making, rather than producing a definitive final decision tree.

### Standard Feature-Outcome Associations

Standard feature-outcome associations in our cohort (reported in details in Supplementary Material) confirmed and extended previously published data showing that patients with abnormal CMCT and/or abnormal SEP show less reduction in dystonia (measured using BFMDRS-m) with pallidal DBS than those without such abnormalities (6). These conclusions are derived from contrasts of the outcome between groups with and without a given feature, or from correlations between features and outcome. However, feature-outcome associations do not provide a measure of the sensitivity and specificity of any derived prediction, nor, in their simplest form, do they lend themselves to the consideration of combinations of features.

### Decision Tree Analysis

We were particularly interested in using this cohort to explore how decision trees can provide clinically useful decision support tools able to inform surgical decisions while at the same time providing estimates of the sensitivity and specificity of the underlying outcome predictions. In effect, these estimates allow the clinician to weigh the significance of each step in the decision tree. The first clear “decision point” in our decision tree analysis is whether the patient has an idiopathic or genetic dystonia vs. an acquired dystonia, as DBS for the former categories is likely to have a favorable outcome. This decision point is consistent with both the literature (see Introduction) and our own feature-outcome association analyses. The second “decision point” suggests that the CMCTs and baseline BFMDRS-m can be used in combination to help decide on whether DBS may have a favorable outcome in patients with acquired dystonia. In this analysis, a normal CMCT and more severe BFMDRS-m suggest that DBS is, on balance, likely to have a positive outcome in acquired dystonia.

However, CMCT is not always available. TMS is not performed in some centers and, even when available it is sometimes contra-indicated, for example in the presence of a cochlear implant. Furthermore, despite best efforts, it may be attempted but without successful data acquisition as a small number of patients do not tolerate the stimulus, especially children with high thresholds. Our ML analyses indicate that where CMCT is not available, SEP alone is still helpful in improving accuracy of outcome prediction, and this forms the basis for an alternative decision tree where SEP status provides the basis for the second decision point. The feature-outcome association analyses previously performed by McClelland et al. (6) and extended here were also able to identify CMCT and SEPs as having predictive value, helping to again validate the DT approach. However, they were not able to assess the predictive value of combined features.

The specific etiology of acquired cases, baseline severity (alone), the results of cranial MRI, and gender were of no additional value at this stage, in deciding whether surgery might be worthwhile, and did not improve upon the majority class classifier when considered in isolation (Note this does not diminish the role of imaging. Cranial MRI already plays an important role in the classification of dystonia as acquired or not, by demonstrating whether structural or degenerative changes are present). BFMDRS-m severity only improved the accuracy with which outcome could be predicted in acquired dystonia when considered in combination with CMCTs, but none of the other non-electrophysiological attributes afforded additional predictive value in combination. Baseline severity did not improve the accuracy of prediction in the smaller analysis performed to reflect the situation where CMCT was not available (Figure 8). The reason for this is not clear, but could be a reflection of the smaller numbers in this analysis.

Previous literature reporting the association between disease severity and DBS outcomes shows conflicting results (7, 31, 32). Moro et al. (31) in a meta-analysis of 523 isolated inherited or idiopathic cases undergoing DBS surgery reported a multivariate meta-regression of absolute BFMDRS-m scores indicating that higher BFMDRS motor and disability scores before surgery, together with younger age at time of surgery, were the main factors associated with significantly better DBS outcomes at the latest follow-up. In contrast, the study by Badhiwala et al. (7) reported that in 125 patients with acquired dystonia (derived from a systematic review and individual patient data meta-analysis), a higher disease severity was associated with poor DBS outcomes. It is important to highlight the key differences between our study and the work by Badhiwala et al. (7) that may explain this difference. Firstly, their work derived latent variables (each such variable is a combination of several features with different weights, estimated in a data-driven manner) and did not assess the contribution of individual features. Secondly, it is possible that the difference in proportion of children with neurodegenerative conditions between our study (<10%) and their study (~40%) may explain this discrepancy (patients with neurodegenerative conditions often have higher baseline severity scores, but will inevitably worsen with time despite an initial response to DBS). Lastly, the latent variable that Badhiwala et al. (7) found to suggest that higher baseline severity leads to poorer DBS outcomes was not only dependent on baseline severity, but several other features (such as age at onset, age at surgery, duration and proportion of life with dystonia). In our case, however, the DT we derived suggests that only in patients with normal CMCT are children with low baseline severity likely to have less favorable DBS outcomes.

Some previous reports (7, 8, 31) have looked at the potential role of age at onset of dystonia or proportion of life lived with dystonia as potential predictive factors for DBS outcome. Age at onset and proportion of life lived with dystonia are inextricably linked with etiology (e.g., those with dystonic cerebral palsy, the largest sub-group of acquired dystonia, have onset in the perinatal period and have therefore spent virtually the whole of their lives with dystonia). These parameters were therefore not chosen for the present analysis because of this potential confound, especially since our work focused on patients with childhood-acquired dystonia.

Unlike the basic feature-outcome associations, the DT analysis requires a threshold to be set for outcome, above which the DT considers a good outcome/favorable prognosis and below which the DT considers a poor outcome/less favorable prognosis. Choosing a threshold is not straightforward, particularly using the BFMDRS-m, with its inherent limitations in this population. A level of 20% improvement in BFMDRS-m has been used in several previous studies, but clinically important changes are still observed in many young people with acquired dystonia, even where this level is not achieved (13, 27, 28, 30). Day to day fluctuations in performance on this scale within a given individual are noted and inter-rater reliability is a further important consideration. Within our own service, inter-rater reliability is ~6–10% (Gimeno unpublished observations). A cut-off point will therefore always, in reality, reflect a range of values and this caveat should be kept in mind when interpreting the findings. The threshold of 0 chosen here has the benefit of showing negative changes (worsening of BFMDRS-m) as a poor outcome and positive changes (any improvement in BFMDRS-m) as a good outcome, which is visually easy to conceptualize. Adjusting the cut-off threshold between 0 and 5% in the current analysis produced comparable results in terms of which factors improved the accuracy above the majority class classifier. Above this level there were insufficient cases classified as having a positive outcome for the ML algorithm to work effectively.

An alternative outcome measure, the COPM, was also assessed in the current study. Patients with abnormal SEPs show a trend towards less benefit in terms of functional goal achievement (COPM) compared with those with normal SEPs (see Supplementary Material). Applying the current DT methods using a threshold of ≥2 point improvement in COPM score did not identify any parameter that performed better than the majority class classifier. The reasons for this are uncertain but could include the smaller number of cases for whom COPM data was available, or the higher proportion of cases that improve with this threshold i.e., there may be insufficient numbers of cases in the “no improvement” group for the ML algorithm to work effectively. The findings could also reflect the nature of the goal-setting process, which takes into account all the clinical assessments in setting realistic and achievable goals. Further objective, blind-rated outcome measures are therefore needed for future work.

Another consideration when assessing outcomes is the potential impact of GPi DBS on non-motor functions. We have evaluated cognitive abilities before and after DBS across the etiological spectrum in childhood dystonia and found overall no adverse consequences of GPi DBS in genetic (33) or acquired dystonias (including dystonic cerebral palsy) (34) or neurodegeneration with brain iron accumulation (NBIA) (35). Recent work also demonstrates the benefit of DBS in reducing pain in children with dystonia and dystonic cerebral palsy (36). Further work is currently ongoing to assess non-motor functioning pre- and post-DBS in more detail.

### Study Strengths

Previous work has shown a significant correlation between the outcome of neurophysiological tests, CMCT and SEP, and change in BFMDRS-m (and for SEP, the change in COPM scores) following DBS (6). However, it was not obvious from that study how much value there might be in basing a decision about DBS surgery on CMCT and SEP results (37). This analysis extends the previous work by using Machine Learning techniques to incorporate these features into a clinical decision support tool, where the recommendations can be assessed through the sensitivity and specificity of the predictions underscoring each step. In addition, a larger sample of patients was available for the current analysis. The study shows how the DT methodology can be used to assess combinations of features (e.g., baseline severity, CMCT, etc.) and convert them into a practical tool that can provide a guide to prognosis for DBS. As these results are based on existing data they therefore represent an evidence-based approach to clinical decision making. This work also quantifies the extent of error to be expected if the proposed decision tree is used, in terms of sensitivity and specificity. It therefore identifies which features should be included in clinical decision making and provides a methodological framework to systematically explore contributions from multiple features. An evidence-based DT and sensitivity/specificity curve might also potentially assist clinical teams when counseling patients and families about expected benefit from DBS. It can therefore help with managing expectations and makes steps toward providing a personalized prognosis, sensitivity and specificity. Indeed, we demonstrate how case-specific preferences, such as the strong wish not to miss the possibility of a positive outcome, can be accommodated within this framework. Lastly, the algorithm developed is highly interpretable and accessible to health care professionals.

### Study Limitations

Firstly, our decision trees are based on numbers of patients that are relatively small for typical machine learning applications that have previously been shown to work well in healthcare with large datasets (38). Nevertheless, the DT method was able to pick out the features that were most predictive of outcome and produce a tree that makes clinical sense, and which is concordant with other analyses. Secondly, the data are all from a single center and we were only able to do an internal validation with k-fold cross validation technique. It is thus possible that the results presented in this work may be subject to overfitting and may not generalize well. Third, this study was a retrospective study and the clinical decision to proceed or not to DBS may have already been influenced by theoretical assumptions relating to neurophysiological results (6) leading to a possible circularity. A further factor is that the analysis here is based on 1-year outcome data. Improvements following DBS continue beyond 1 year and even up to 5 years, so future work is needed to assess whether the DTs developed here will still help in predicting these later improvements. Lastly, the outcomes in this study were based primarily on changes in the BFMDRS-m. This scale has significant limitations for childhood-onset dystonia and acquired dystonia and is not sensitive to some changes which are still meaningful for young people and their families (13, 27). We are working towards obtaining data with other objective outcome scales, but numbers are not yet sufficient to apply a ML method to these data. Preliminary data (Gimeno and McClelland, unpublished observations) indicate that patients with abnormal neurophysiological tests show less benefit from DBS as measured using other such scales, but this requires confirmation by further analysis with larger subject numbers.

## Conclusion

This study is the first exploration of how a ML-based approach could be used to predict potential benefit from DBS in children with dystonia and to aid clinical decision making and counseling of families about expected outcome. Although ML methods generally excel in very large datasets, the Decision Tree methodology provides a data-driven, highly interpretable, example decision support tool even in our modestly sized cohort. The key finding is that neurophysiological parameters can help to predict the outcome of DBS. We encourage other centers to introduce neurophysiological measures to their assessment pathways and to collect data to facilitate future multi-center evaluation of these potential predictive markers and the testing of the illustrative decision support tools presented here. Future work will also consider additional outcome measures and thereby broaden DT-based decision support tools.

## Data Availability Statement

The code of our analysis is available at https://github.com/syedahmar/ChildrenDystoniaDBS/tree/master. The data analyzed in this study is subject to the following licenses/restrictions: De-identified data will be made available to interested researchers upon scientific protocol evaluation committee approval and subject to study governance structure and patient consent form, following a time restriction. Requests for data access can be submitted to Verity M. McClelland. Note the data management plan approved by the funders states the following: The research team will have exclusive use of the data for the project duration and 3 years afterwards to allow for publications to be achieved. After this period, the majority of data would be made available for data sharing via the King's College London data repository. Requests to access these datasets should be directed to verity.mcclelland@kcl.ac.uk.

## Ethics Statement

The studies involving human participants were reviewed and approved by London-Harrow National Research Ethics Committee, London, UK (17/LO/0439). Written informed consent from the participants' legal guardian/next of kin was not required to participate in this study in accordance with the national legislation and the institutional requirements.

## Author Contributions

PB with VM conceived the initial idea. SS led and undertook the development of the machine learning methodology and data analysis. VM curated and analyzed the neurophysiological data. SS wrote the initial draft of the paper and all co-authors contributed to and approved the final version of the manuscript. J-PL created and leads the Evelina London Children's Hospital Complex Motor Disorders Service for deep brain stimulation neuromodulation, initiated measuring the Central Motor Conduction Time (CMCT) and Somatosensory Evoked Potentials (SEP) as an extension to the clinical phenotype of children with dystonia and other movement disorders.

## Conflict of Interest

J-PL has received unrestricted educational support for instructional courses and consultancy fees from Medtronic Ltd. The remaining authors declare that the research was conducted in the absence of any commercial or financial relationships that could be construed as a potential conflict of interest.
