# Supplementary material for: Application of Machine Learning Using Decision Trees for Prognosis of Deep Brain Stimulation of Globus Pallidus Internus for Children With Dystonia
Source: Front Neurol. 2020 Aug 14;11:825. doi: 10.3389/fneur.2020.00825 (PMC7115974; doi:10.3389/fneur.2020.00825)
Supplement: Supplementary file 1 [file Data_Sheet_1.DOCX]

# Supplementary Data

# Application of Machine Learning Using Decision Trees for Prognosis of Deep Brain Stimulation of Globus pallidus internus for Children with Dystonia

1. **Central Motor Conduction Time (CMCT) results**

Of 233 children tested, technically satisfactory CMCT data were obtained from 191 children. CMCTs were abnormal (prolonged CMCT or absent MEP) in at least one limb in 34 (19.3%) children. The distribution of children with abnormal CMCTs across the aetiological groups is shown in Fig S-1. Abnormal CMCTs were seen in a higher proportion of children with acquired (20.3%) than idiopathic/genetic dystonia (9.3%). Of the idiopathic/genetic group, abnormal CMCTs were seen only in complex idiopathic/genetic dystonia and were not seen in isolated idiopathic/genetic dystonia.

1. **Somatosensory Evoked Potentials (SEP) results**

Of 162 children tested, technically satisfactory SEP data were obtained from 155 children, Fewer children had SEP data than CMCT because SEP testing was introduced to the assessment regimen more recently. An abnormal SEP (cortical response absent, delayed or of abnormal waveform) was found in 63 (40.1%) children. The distribution of children with abnormal SEPs by aetiology is shown in Fig S-1. Abnormal SEPs were seen more often in acquired (45.1%) than idiopathic/genetic dystonia (24.2%) and were seen most frequently in children with dystonic-dyskinetic cerebral palsy and in those with acquired degenerative dystonia (due to Neuronal Degeneration with Brain Iron Accumulation or mitochondrial disease).

**Figure S-1.** Distribution of abnormal neurophysiology results across aetiological groups. Bars show percentage of children in each aetiological group with one or more abnormal cortical SEP (grey) or CMCT result (red). Note different denominators: CMCT abnormal in 0/18 Genetic/Idiopathic Isolated, 4/25 Idiopathic Complex, 15/82 Acquired perinatal CP, 1/ 19 Acquired metabolic, 10/33 Acquired non-degenerative ‘‘Other” and 4/14 Acquired degenerative. SEP abnormal in 1/12 Genetic/Idiopathic Isolated, 7/21 Idiopathic Complex, 35/70 Acquired perinatal CP, 4/16 Acquired metabolic, 10/29 Acquired non-degenerative ‘‘Other” and 6/7 Acquired degenerative patients.

1. **Cranial MRI findings**

Cranial MRI scans were classified by the pattern of findings, in keeping with previous publications, as follows:

|  | **Classification of radiological findings** | **Abbreviation for figure** |
| --- | --- | --- |
| 1 | Normal | Normal |
| 2 | Periventricular white matter change VWM | PVWM |
| 3 | Basal Ganglia including Globus pallidus | BG-GP |
| 4 | Basal Ganglia + posterior white matter | BG + Post WM |
| 5 | Basal Ganglia + Peri-rolandic cortex + caudate + thalamus (typical picture of term hypoxic ischaemic encephalopathy) | BG + Peri-rolandic + caudate/Thal |
| 6 | Miscellaneous changes involving basal ganglia (not globus pallidus), thalamus or cortex | Misc (BG or Thal ) |
| 7 | Miscellaneous changes not involving basal ganglia (eg prominent lateral ventricles, cerebellar abnormalities, minor hypomyelination and other miscellaneous abnormalities | Misc (non-BG/ Thal) |

Table S-1.

The distribution of children with abnormal CMCT and SEP in relation to cranial MRI is shown in Fig S-2. Abnormal CMCT and SEPs were identified in children across all imaging categories, including those with normal MRI, of whom 10/40 (25%) had abnormal SEPs and 6/48 (12.5%) had abnormal CMCT. Abnormal SEPs and CMCT were most frequently present in the children with evidence of periventricular white matter damage on MRI.

**Fig. S-2.** Distribution of abnormal neurophysiology results across Clinical MRI Diagnostic Categories. Bars show percentage of children in each imaging group with one or more abnormal cortical SEP (grey) or CMCT result (red). Imaging abnormality categories defined as in Table S-1 above.

1. **Standard Statistical comparisons of outcome between groups with normal and abnormal neurophysiological parameters**

**CMCT data**

111 children with satisfactory CMCT data went forward for DBS and had 1 year outcome data available. Those with abnormal CMCT (n=16) had significantly less improvement on the BFMDRS-m scale at 1 year compared with those with normal CMCT (n=95) (Mann-Whitney U = 370, p=0.001) (Fig S-3A).

**SEP data**

77 children with satisfactory SEP data went forward for DBS and had 1 year outcome data available. Those with abnormal SEP (n=27) had significantly less improvement on the BFMDRS-m scale at 1 year compared with those with normal SEP (n=50) (Mann-Whitney U = 378, p=0.002) (Fig. S-3B).

**Fig. S-3.** Tukey Box-plots showing % improvement in BFMDRS-m score at 1 year post-DBS for (A) patients with normal/abnormal CMCT and (B) patients with normal/abnormal SEP. Tukey Box-Plots shows median and interquartile range for each group, whiskers show 75^th^ centile plus 1.5IQR and 25^th^ centile minus 1.5 IQR. Outliers beyond these points are shown as individual values. p values show results of Mann-Whitney test.

To address possible confounding and to focus on the acquired dystonia group, sub-group analysis was performed. Figure S-4 shows the sub-group data for the Genetic/Idiopathic group (A-B) and the Acquired group (C-D). Patient numbers in the primary group with abnormal CMCT or abnormal SEP were small, so statistical testing is only presented for the acquired group. For the Acquired Group, those with abnormal CMCT (n=14) had significantly less improvement than those with normal CMCT (n=64) (Mann-Whitney U = 226.0, p=0.004) (Fig. S-3C) and those with abnormal SEP (n=23) had significantly less improvement than those with normal SEP (n=31) (Mann-Whitney U = 237.0, p=0.037) (Fig. S-4C).

**Fig. S-4.** Tukey Box-plots showing % improvement in BFMDRS-m score at 1 year post-DBS for patients with genetic/idiopathic dystonia (top row) and patients with acquired dystonia (bottom row). Plots are shown separately for (A + C) patients with normal/abnormal CMCT and (B + D) patients with normal/abnormal SEP. Details for Tukey Box-Plots as in Fig S-3. Note different scales as improvements are generally smaller in acquired dystonia.

1. **COPM Data**

COPM scores at 1 year post DBS were available for 70 patients with technically satisfactory CMCT data and 62 patients with technically satisfactory SEP data. The COPM-p (performance scale) as rated by the parents was used, except in nine teenagers in whom the child’s own score was used, as this was the only score available.

There was a trend towards lower change in COPM-p scores at one year post DBS in patients with abnormal CMCT than those with normal CMCT but this did not reach statistical significance (normal CMCT: N=62, mean 2.702, SD 2.092; abnormal CMCT N=8 mean 2.106, SD 0.568) t = 1.789 p = 0.081.

Patients with abnormal SEP also tended to show lower COPM-p scores at one year post DBS than those with normal SEP. For patients undergoing DBS prior to June 2016 (when relationship between neurophysiological parameters and outcome was first observed) mean values were as follows: normal SEP N=43, mean 3.206, SD 2.124; abnormal SEP N=19 mean 2.045, SD 1.356) and this difference was statistically significant (t=2.585, p=0.013) similar to the previous cohort (McClelland et al., 2018).

*With inclusion of more recent patients, mean values are as follows: normal SEP N=50, mean 3.105, SD 2.016; abnormal SEP N=21, mean 2.345, SD 1.659 and the statistical significance is lost (t=1.522, p=0.133).*

*The reason for this is unclear. However, it is noted that goal setting for the COPM is done just prior to surgery and takes into account all the pre-operative clinical assessments, including imaging and neurophysiology, as well as patient and family preferences. As noted previously, theoretical assumptions and implications regarding abnormal CMCT/SEP results will have influenced both the decision to proceed or not to DBS and also the counselling of families regarding benefit (McClelland et al., 2018). This may explain the lack of a difference in COPM scores in relation to CMCT findings seen in the previous analysis and may also contribute to the loss of significance in COPM scores in relation to normal/abnormal SEP that is seen on inclusion of more recent patients in the cohort.*
